# Effects of preselection of genotyped animals on reliability and bias of genomic prediction in dairy cattle

**DOI:** 10.5713/ajas.18.0161

**Published:** 2018-07-26

**Authors:** Kenji Togashi, Kazunori Adachi, Kazuhito Kurogi, Takanori Yasumori, Kouichi Tokunaka, Atsushi Ogino, Yoshiyuki Miyazaki, Toshio Watanabe, Tsutomu Takahashi, Kimihiro Moribe

**Affiliations:** 1 Maebashi Institute of Animal Science, Livestock Improvement Association of Japan, Maebashi, Gunma 371-0121, Japan; 2 Livestock Improvement Association of Japan, Koto-ku, Tokyo 135-0041, Japan

**Keywords:** Reliability of Selection, Genomic Selection, Reference Population, Dairy Cattle

## Abstract

**Objective:**

Models for genomic selection assume that the reference population is an unselected population. However, in practice, genotyped individuals, such as progeny-tested bulls, are highly selected, and the reference population is created after preselection. In dairy cattle, the intensity of selection is higher in males than in females, suggesting that cows can be added to the reference population with less bias and loss of accuracy. The objective is to develop formulas applied to any genomic prediction studies or practice with preselected animals as reference population.

**Methods:**

We developed formulas for calculating the reliability and bias of genomically enhanced breeding values (GEBV) in the reference population where individuals are preselected on estimated breeding values. Based on the formulas presented, deterministic simulation was conducted by varying heritability, preselection percentage, and the reference population size.

**Results:**

The number of bulls equal to a cow regarding the reliability of GEBV was expressed through a simple formula for the reference population consisting of preselected animals. The bull population was vastly superior to the cow population regarding the reliability of GEBV for low-heritability traits. However, the superiority of reliability from the bull reference population over the cow population decreased as heritability increased. Bias was greater for bulls than cows. Bias and reduction in reliability of GEBV due to preselection was alleviated by expanding reference population.

**Conclusion:**

Cows are easier in expanding reference population size compared with bulls and alleviate bias and reduction in reliability of GEBV of bulls which are highly preselected than cows by expanding the cow reference population.

## INTRODUCTION

Genomic prediction (GP) is used to predict the genomic breeding values of genotyped individuals [1]. The GP models usually do not account for selection. However, the reference population which is used for estimating marker effects with GP models usually consisted of progeny test bulls which was highly selected. Therefore, the prediction models are unable to incorporate past selection based on pedigree and phenotypes, perhaps leading to bias as well as decreased accuracy.

A formula for approximating the reliability and bias of the genomically enhanced breeding values (GEBV) that accounted for the prior selection of genotyped test bulls from among all test bull candidates was proposed [2]. In that method, the differences between the means and standard deviations of the estimated breeding values (EBV) of all of the test bull candidates are used to estimate the proportion of selective genotyping. Then, the selection difference or intensity of selection is calculated from quantitative genetics textbooks [3], and the authors approximated the reliability and bias of the GEBV by accounting for the effect of the intensity of selection [2]. However, the true genetic variance was reduced not only by the intensity of selection but also by the reliability of EBV [3]. The reliability of EBV differs by trait and between males and females. In dairy cattle populations, the intensity of selection is higher in males than in females [4], suggesting that cows potentially could be added to the reference population with less bias and loss of accuracy.

The genotyping of cows has become more prevalent as the cost of genotyping, in general, has decreased. The same individuals should be both genotyped and phenotyped, instead of genotyping the parents and phenotyping their progeny [5]. Adding genotyped females and their phenotypic records to the existing sire reference population is expected to increase the reliability of GPs, and increase in reliability would lead to increase in genetic gain and decreasing inbreeding in dairy cattle [6].

The genetic correlation between phenotypes of bulls and cows was approximately 0.6 for all yield traits and differed significantly from 1 [7]. Using selection index theory, the reliability of GEBV for a reference population in which the information contents in their phenotypes differed between groups, i.e., a reference population consisting of sires and cows both was presented [8]. However, in that method, the effect of preselection on reliability was not taken into account. Revaluation of cows would be beneficial from the standpoints of their less intense selection and easier incorporation into the reference population for its expansion, especially from the standpoints based on the bias and accuracy of GPs by varying the magnitudes of heritability and intensity of preselection among the animals chosen to create the reference population. A deterministic prediction model is necessary to develop simple formulas for calculating the reliability and bias of GEBV that accounts for prior selection of animals in the reference population. There are some results about using cows in the reference population with real data [9–12]. Construction of the real mixed reference population based on the deterministic model presented would improve the reliability of GEBV.

The first objective of the current study was to develop formulas for calculating the reliability and bias of GPs in which the effects of both intensity of selection and the reliability of EBV of preselected animals in the reference population would be taken into account. Next is to present a formula to calculate the number of bulls equal to a cow in regard to creating the same reliabilities of the GEBV between preselected bulls and cows in the reference population. The last is to present a guideline to create a reference population composed of preselected bulls and cows to prevent the reduction of reliability and bias of GEBV due to preselection before the actual creation of the reference population.

## MATERIALS AND METHODS

### Formulas for reliability and bias under selection

The variance of true breeding value of the animals selected on the basis of EBV can be expressed as:

$$\sigma_{{\text{G}}^{*}}^{2}=\sigma_{\text{G}}^{2}(1-{\text{kr}}_{\text{EBV}}^{2}),$$

where * denotes values after selection, G is true breeding value, $\sigma_{\text{G}}^{2}$is the variance of G before selection, k is the proportional reduction in the variance of the selection criterion due to selection, and ${\text{r}}_{\text{EBV}}^{2}$ is the reliability of EBV. With truncation selection on a normally distributed selection criterion, k is determined entirely by the intensity of selection, k = i (i − x), where i is the intensity of selection, and x is the standardized truncation point [3]. Similarly, the variance of the GEBV of the animals selected on the basis of EBV can be expressed as:

$$\sigma_{{\text{GEBV}}^{*}}^{2}=\sigma_{\text{GEBV}}^{2}(1-{\text{kr}}_{\text{EBV},\text{GEBV}}^{2}),$$

where $\sigma_{\text{GEBV}}^{2}$ is the variance of the GEBV before selection, and ${\text{r}}_{\text{EBV},\text{GEBV}}^{2}$ is the squared correlation between EBV and GEBV.

Assuming prediction error cov(EBV, GEBV) is zero, the correlation between EBV and GEBV can be shown as:

$${\text{r}}_{\text{EBV},\text{GEBV}}={\text{r}}_{\text{EBV}}{\text{r}}_{\text{GEBV}}.$$

Therefore, the variance of the GEBV after selection is:

$$\sigma_{{\text{GEBV}}^{*}}^{2}=\sigma_{\text{GEBV}}^{2}(1-{\text{kr}}_{\text{EBV},\text{GEBV}}^{2})=\sigma_{\text{GEBV}}^{2}(1-{\text{kr}}_{\text{EBV}}^{2}{\text{r}}_{\text{GEBV}}^{2}).$$

In addition, the cov(EBV, GEBV) can be written as:

$$\sigma_{\text{EBV},\text{GEBV}}={\text{r}}_{\text{EBV}}{\text{r}}_{\text{GEBV}}\sigma_{\text{EBV}}\sigma_{\text{GEBV}}={\text{r}}_{\text{EBV}}^{2}{\text{r}}_{\text{GEBV}}^{2}\sigma_{\text{G}}^{2}.$$

A general expression for a covariance after selection [13] is:

$$\sigma_{\text{jk}}^{*}=\sigma_{\text{jk}}-\text{k}\frac{\sigma_{\text{ij}}\sigma_{\text{ik}}}{\sigma_{\text{i}}^{2}},$$

where $\sigma_{\text{jk}}^{*}$ is the covariance between j and k selected on i, and σ_jk_ is the covariance before selection. Therefore the cov(GEBV, G) after selection on EBV is:

$$\begin{array}{l} \sigma_{\text{G},{\text{GEBV}}^{*}}=\sigma_{\text{G},\text{GEBV}}-\text{k}\frac{\sigma_{\text{G},\text{EBV}}\sigma_{\text{GEBV},\text{EBV}}}{\sigma_{\text{EBV}}^{2}}\\ =\sigma_{\text{GEBV}}^{2}-\text{k}\sigma_{\text{GEBV},\text{EBV}}=\sigma_{\text{GEBV}}^{2}-{\text{kr}}_{\text{EBV}}^{2}{\text{r}}_{\text{GEBV}}^{2}\sigma_{\text{G}}^{2}\\ =\sigma_{\text{GEBV}}^{2}(1-{\text{kr}}_{\text{EBV}}^{2}). \end{array}$$

In summary, the reliability of GEBV after accounting for selection on EBV can be expressed based on the reliability of GEBV under random selection (${\text{r}}_{\text{GEBV}}^{2}$), the intensity of selection, and the reliability of EBV (${\text{r}}_{\text{EBV}}^{2}$) of the preselected individuals both genotyped and phenotyped in the reference population:

(1) $$\begin{array}{l} {\text{r}}_{\text{GEBV},{\text{G}}^{*}}^{2}=\frac{\sigma_{\text{G},\text{GEBV}*}^{2}}{\sigma_{\text{G}*}^{2}\sigma_{\text{GEBV}*}^{2}}=\frac{\sigma_{\text{GEBV}}^{2}(1-{\text{kr}}_{\text{EBV}}^{2})}{\sigma_{\text{G}}^{2}(1-{\text{kr}}_{\text{EBV}}^{2}{\text{r}}_{\text{GEBV}}^{2})}\\ ={\text{r}}_{\text{GEBV}}^{2}\frac{1-{\text{kr}}_{\text{EBV}}^{2}}{1-{\text{kr}}_{\text{EBV}}^{2}{\text{r}}_{\text{GEBV}}^{2}} \end{array}$$

When ${\text{r}}_{\text{EBV}}^{2}=1$, equation (1) yields the same formula as that in [2].

The regression coefficient of G or deregressed proofs on GEBV is a criterion for bias in GEBV [2,14,15]. Accordingly, the regression coefficient of G on GEBV after selection by using EBV is written as:

(2) $$\begin{array}{l} \text{b}*=\frac{\sigma_{\text{G},\text{GEBV}*}}{\sigma_{\text{GEBV}*}^{2}}=\frac{\sigma_{\text{GEBV}}^{2}(1-{\text{kr}}_{\text{EBV}}^{2})}{\sigma_{\text{GEBV}}^{2}(1-{\text{kr}}_{\text{EBV}}^{2}{\text{r}}_{\text{GEBV}}^{2})}\\ =\frac{1-{\text{kr}}_{\text{EBV}}^{2}}{1-{\text{kr}}_{\text{EBV}}^{2}{\text{r}}_{\text{GEBV}}^{2}} \end{array}$$

When ${\text{r}}_{\text{EBV}}^{2}=1$, equation (2) results in the same regression coefficient as in [2]. That is, we extended the formula for the reliability and bias of GEBV from [2] by accounting for the preselection on EBV of the animals used to create the reference population.

### Estimating the number of bulls equal to a cow in the reliability of the GEBV of preselected animals in the reference population

Reliability in a reference population after selection was expressed as (1). The reliability of GEBV without selection (${\text{r}}_{\text{GEBV},\text{G}}^{2}$) or under random selection is obtained after transformation of (1):

(3) $${\text{r}}_{\text{GEBV},\text{G}}^{2}=\frac{{\text{r}}_{\text{GEBV},\text{G}*}^{2}}{1-{\text{kr}}_{\text{EBV}}^{2}+{\text{kr}}_{\text{GEBV},\text{G}*}^{2}{\text{r}}_{\text{EBV}}^{2}}$$

The reliabilities of GEBVs depend on the size of the reference population (nP), the effective number of loci for which effects have to be estimated (nG), and the correlation of the G of a genotyped individual with its phenotypic record (r). In a random sample of the population, the reliability of GEBV or the correlation between GEBV and G (${\text{r}}_{\text{GEBV},\text{G}}^{2}$) can be calculated as described in [16]:

(4) $${\text{r}}_{\text{GEBV},\text{G}}^{2}=\frac{\lambda {\text{r}}^{2}}{\lambda {\text{r}}^{2}+1}$$

where λ = nP/nG. Parameter nG depends on the historical effective size of the unselected population (N_E_) and on the size of the genome, L (in Morgans), and can be estimated as shown in [17]:

$$\text{nG}=2{\text{N}}_{\text{E}}\text{L}$$

When an individual in the reference population is both genotyped and phenotyped, r is equal to the square root of heritability of the trait. Then, the reliability of cows from their own records is:

$${\text{r}}^{2}={\text{h}}^{2}$$

When the reference population is based on progeny-tested sires, i.e., when sires are genotyped but their offspring are phenotyped, r equals the accuracy of the EBV obtained from progeny testing [5]:

$${\text{r}}^{2}=\frac{0.25{\text{Nh}}^{2}}{1+0.25(\text{N}-1){\text{h}}^{2}},$$

where N is the number of half-sibling progeny on which the EBV is based.

Parameter nP can be transformed from (4):

(5) $$\text{nP}=\text{nG}\frac{{\text{r}}_{\text{GEBV},\text{G}}^{2}}{{\text{r}}^{2}(1-{\text{r}}_{\text{GEBV},\text{G}}^{2})}$$

When the reference population is composed of either bulls (m) or cows (f), parameter nP in (5) can be written as:

$$\begin{array}{l} {\text{nP}}_{\text{m}}=\text{nG}\frac{{\text{r}}_{\text{m}\_\text{GEBV},\text{G}}^{2}}{{\text{r}}_{\text{m}}^{2}(1-{\text{r}}_{\text{m}\_\text{GEBV},\text{G}}^{2})}\;\text{or}\\ {\text{nP}}_{\text{f}}=\text{nG}\frac{{\text{r}}_{\text{f}\_\text{GEBV},\text{G}}^{2}}{{\text{r}}_{\text{f}}^{2}(1-{\text{r}}_{\text{f}\_\text{GEBV},\text{G}}^{2})}, \end{array}$$

where the subscript letters m and f refer to male and female, respectively.

Therefore,

(6) $$\frac{{\text{nP}}_{\text{m}}}{{\text{nP}}_{\text{f}}}=\frac{{\text{r}}_{\text{f}}^{2}(1-{\text{r}}_{\text{f}\_\text{GEBV},\text{G}}^{2}){\text{r}}_{\text{m}\_\text{GEBV},\text{G}}^{2}}{{\text{r}}_{\text{m}}^{2}(1-{\text{r}}_{\text{m}\_\text{GEBV},\text{G}}^{2}){\text{r}}_{\text{f}\_\text{GEBV},\text{G}}^{2}}$$

Alternatively, the reliability of GEBV under random selection can be written as (3) by using that of GEBV after preselection, therefore using the subscript letters m and f as defined earlier,

$${\text{r}}_{\text{m}\_\text{GEBV},\text{G}}^{2}=\frac{{\text{r}}_{\text{m}\_\text{GEBV},\text{G}*}^{2}}{1-{\text{k}}_{\text{m}}{\text{r}}_{\text{m}\_\text{EBV}}^{2}+{\text{k}}_{\text{m}}{\text{r}}_{\text{m}\_\text{GEBV},\text{G}*}^{2}{\text{r}}_{\text{m}\_\text{EBV}}^{2}}$$

and

(7) $${\text{r}}_{\text{f}\_\text{GEBV},\text{G}}^{2}=\frac{{\text{r}}_{\text{f}\_\text{GEBV},\text{G}*}^{2}}{1-{\text{k}}_{\text{f}}{\text{r}}_{\text{f}\_\text{EBV}}^{2}+{\text{k}}_{\text{f}}{\text{r}}_{\text{f}\_\text{GEBV},\text{G}*}^{2}{\text{r}}_{\text{f}\_\text{EBV}}^{2}}$$

Substituting (7) into (6) yields:

(8) $$\frac{{\text{nP}}_{\text{m}}}{{\text{nP}}_{\text{f}}}=\frac{{\text{r}}_{\text{f}}^{2}}{{\text{r}}_{\text{m}}^{2}}\times \frac{{\text{r}}_{\text{m}\_\text{GEBV},\text{G}*}^{2}}{{\text{r}}_{\text{f}\_\text{GEBV},\text{G}*}^{2}}\times \frac{\left(1-{\text{k}}_{\text{f}}{\text{r}}_{\text{f}\_\text{EBV}}^{2})(1-{\text{r}}_{\text{f}\_\text{GEBV},\text{G}*}^{2}\right)}{\left(1-{\text{k}}_{\text{m}}{\text{r}}_{\text{m}\_\text{EBV}}^{2})(1-{\text{r}}_{\text{m}\_\text{GEBV},\text{G}*}^{2}\right)}$$

The numbers of bulls equal to a cow in regard to the specific reliabilities of the GEBV of animals in the reference population with and without preselection are calculated by using (8) and (6), respectively. Note that ${\text{r}}_{\text{f}\_\text{EBV}}^{2}$ and ${\text{r}}_{\text{m}\_\text{EBV}}^{2}$ are the reliabilities of EBV for preselection used to create the females-only and males-only reference population, respectively. The number of bulls equal to a cow in terms of the reliability of GEBV must be compared under the same reliability of GEBV after preselection, i.e., ${\text{r}}_{\text{m}\_\text{GEBV},\text{G}*}^{2}={\text{r}}_{\text{f}\_\text{GEBV},\text{G}*}^{2}$.

Therefore, the number of bulls equal to a cow in a standpoint of bringing about the same size of reliabilities of the GEBV of preselected animals in the reference population is:

(9) $$\frac{{\text{nP}}_{\text{m}}}{{\text{nP}}_{\text{f}}}=\frac{{\text{r}}_{\text{f}}^{2}}{{\text{r}}_{\text{m}}^{2}}\times \frac{\left(1-{\text{k}}_{\text{f}}{\text{r}}_{\text{f}\_\text{EBV}}^{2}\right)}{\left(1-{\text{k}}_{\text{m}}{\text{r}}_{\text{m}\_\text{EBV}}^{2}\right)}$$

Note that the number of bulls equal to a cow in terms of the reliability of GEBV without preselection, i.e., k = 0, depends only on the reliability of EBV of the individuals both genotyped and phenotyped in the reference population.

### Reliability of GEBV in the reference population consisting of preselected bulls and cows

Using selection index theory, the reliability of GEBV was derived by [10], which is explained by markers, in a reference population consisting of multiple groups of animals whose phenotypes differ in their information content. We extended selection index theory to a reference population consisting of preselected bulls and cows.

From selection index theory,

(10) $$\begin{array}{l} \left[\begin{array}{cc} r_{m\_GEBV,G^{*}}^{2} & r_{m\_GEBV,G^{*}}^{2}r_{f\_GEBV,G^{*}}^{2}\\ r_{f\_GEBV,G^{*}}^{2}r_{m\_GEBV,G^{*}}^{2} & r_{f\_GEBV,G^{*}}^{2} \end{array}\right]\;\left[\begin{array}{c} b_{1}\\ b_{2} \end{array}\right]=\left[\begin{array}{c} r_{m\_GEBV,G^{*}}^{2}\\ r_{f\_GEBV,G^{*}}^{2} \end{array}\right]\\ r_{m+f^{*}}^{2}=b_{1}r_{m\_GEBV,G^{*}}^{2}+b_{2}r_{f\_GEBV,G^{*}}^{2} \end{array}$$

where ${\text{r}}_{\text{m}+\text{f}*}^{2}$ is the reliability of GEBV in the reference population consisting of preselected bulls and cows.

The increase in reliability from including bulls only to both bulls and cows in a reference population is expressed as the difference between the reliability of GEBV in the reference population consisting of preselected bulls and cows and that of including preselected bulls only, i.e., ${\text{r}}_{\text{m}+\text{f}*}^{2}-{\text{r}}_{\text{m}\_\text{GEBV},\text{G}*}^{2}$. This increase in reliability corresponds to the increase after preselection and therefore can be converted to the increase under random selection. That is, the increase (${\text{r}}_{\Delta \_\text{m}+\text{f}}^{2}$) in the reliability of GEBV under random selection due to the addition of cows into the reference population relative to the reference population containing males only can be expressed applying (3):

(11) $${\text{r}}_{\Delta \_\text{m}+\text{f}}^{2}=\frac{{\text{r}}_{\text{m}+\text{f}*}^{2}-{\text{r}}_{\text{m}\_\text{GEBV},\text{G}*}^{2}}{1-{\text{k}}_{\text{m}}{\text{r}}_{\text{m}\_\text{EBV}}^{2}+{\text{k}}_{\text{m}}{\text{r}}_{\text{m}\_\text{EBV}}^{2}({\text{r}}_{\text{m}+\text{f}*}^{2}-{\text{r}}_{\text{m}\_\text{GEBV},\text{G}*}^{2})}$$

The number of bulls corresponding to this increase (nP_m−Δ_) can be computed applying (5):

$${\text{nP}}_{\text{m}-\Delta }=\text{nG}\frac{{\text{r}}_{\Delta \_\text{m}+\text{f}}^{2}}{{\text{r}}_{\text{m}}^{2}(1-{\text{r}}_{\Delta \_\text{m}+\text{f}}^{2})}$$

We designated the number of cows in the reference population as nP_f_. The increase in reliability is derived from adding cows into the reference population that consists of bulls only. That is, the number of bulls equal to a cow in regard to the reliability of GEBV in the reference population consisting of preselected bulls and cows (Cow_value_m+f_) is:

(12) $${\text{Cow}}_{\text{value}\_\text{m}+\text{f}}=\frac{{\text{nP}}_{\text{m}-\Delta }}{{\text{nP}}_{\text{f}}}=\frac{\text{nG}\times {\text{r}}_{\Delta \_\text{m}+\text{f}}^{2}}{{\text{nP}}_{\text{f}}\times {\text{r}}_{\text{m}}^{2}(1-{\text{r}}_{\Delta \_\text{m}+\text{f}}^{2})}$$

### Simulation data

We preselected animals in the reference population according to the EBV of the trait of interest rather than selecting them randomly, to obtain more realistic reference populations [14, 18,19]. The animals in the reference population came from several generations in the past but were approximated and simplified to come from a single generation, i.e., they were preselected on EBV, and the reference population was created from the phenotypic data of the preselected bulls’ daughters’ records or the preselected cows’ own data. When the reference population is based on progeny-tested bulls, the number of daughters per test bull was set to 50 and 100. All test bull candidates were assumed to be preselected according to the PA (parent average). PA was computed by using the EBVs of sire (from 50 and 100 daughters) and dam. When the number of daughters per progeny-tested bull was set to 50, PA was calculated from EBV of sire from 50 daughters. That is, same number was set to the number of daughters of sire in PA and that of progeny-tested bull. Note that bulls for progeny testing were preselected from all test bull candidates, and they became test bulls after preselection and progeny-tested sires with their daughters’ records (50 or 100) after progeny-testing. Heifers were preselected using their PA, and cows were preselected according to the EBV from their own records. After preselection, test bulls, heifers, and cows were used to create the reference population. The reliability of the EBV from their own records was calculated by selection index theory where reliabilities of PA and their individual records constituted the index similar to the equation (10) and the number of daughters of sire in PA was set to 50.

We assumed that the length of the genome was 30 Morgans and that the heritability of the trait of interest was 0.1, 0.3, or 0.5. The historical effective population size was set to 100 animals [5,20]. The preselection percentage on EBV of animals used to create the reference population was set to 5%, 30%, and 100% for males and to 70%, 90%, and 100% for females. When animals were selected randomly, the proportional reduction (k) in the variance of G was set to zero. The reference population size was set to 5,000, 10,000, 20,000, and 40,000.

## RESULTS

### Reliability of GEBV of preselected animals in the reference population

We calculated the reliability of the GEBV of non-preselected animals and the ratio of the reliability of preselected animals to that of non-preselected animals for reference populations composed solely of proven bulls preselected on PA computed by using EBVs of sire (from 50 or 100 daughters) and dam (Table 1). The reliability of the GEBV of cows was shown in Table 2. The reliability of preselection on PA, EBV from a cow’s own record, or a bull’s progeny testing based on 50 daughters was calculated at three levels of heritability (Table 3). The reliability of GEBV in the bulls-only reference population was the highest among the three reference populations (bulls preselected on PA, heifers preselected on PA, and cows preselected on EBV from their own records). The bulls-only population was particularly superior to the cow population for low-heritability traits (h^2^ = 0.1), especially for the bulls-only population testing based on 100 daughters. However, the superiority of the reliability associated with the bull reference population decreased as heritability increased, regardless of whether the animals in the reference population were preselected or not.

In addition, the reliability of GEBV decreased as the intensity of preselection increased (i.e., a decrease in the preselection percentage), and this trend became more conspicuous as heritability increased. This change occurs because the effect of preselection on the reduction of the variance of G increases as heritability increases. The decrease in the reliability of preselected animals compared with that of non-preselected animals became more conspicuous as the reference population size decreased. That is, the effect of preselection on the decrease in reliability became more deleterious as the reference population became smaller.

### Bias of GEBV

Regression coefficients of G on GEBV for animals in the reference populations composed solely of proven bulls preselected on PA calculated by using EBVs of sire (from 50 and 100 daughters) and dam were shown (Table 4). The regression coefficients of G on GEBV for cows preselected on EBV were calculated (Table 5). Regression coefficients of G on GEBV deviated more from 1 as the intensity of preselection increased, thus indicating that overestimation of GEBV became more prominent as the intensity of preselection increased. Because the intensity of preselection is higher in bulls than in cows, bias was more problematic in bulls than in cows. When the cow reference population preselected on PA was compared with that preselected by using the EBV from their own records, bias or overestimation of GEBV was greater for cows preselected on the EBV from their own records than those preselected on PA because the reliability of EBV from their individual record was greater than that of PA (Table 3). In the same way, bias or overestimation of GEBV was greater for bulls testing 100 daughters than 50 daughters. That is, bias became more pronounced with an increase in the reliability of preselection of animals used to create the reference population. Bias or overestimation of GEBV was alleviated by increasing reference population size (Tables 4, 5).

### The contribution to the same reliability of the number of bulls to a cow

The number of bulls equal to a cow in terms of the bringing about the same size of reliability of the GEBV of preselected animals was calculated by (9) (Table 6). This parameter is related solely to the reliability (${\text{r}}_{\text{f}\_\text{EBV}}^{2},{\text{r}}_{\text{m}\_\text{EBV}}^{2}$) of the preselection of heifers/cows and bulls to create the reference population, the intensity of preselection (k_f_, k_m_), and the reliability of the EBV of cow’s record or bull’s progeny testing (${\text{r}}_{\text{f}}^{2},{\text{r}}_{\text{m}}^{2}$). The number of bulls equal to a cow in regard to the reliability increased with increases in the intensity of preselection for males and with decreases in the intensity of selection for females. The number increased three to four times with an increase in heritability from 0.1 to 0.5 under the same preselection percentage. For example, the number of bulls equal to a cow increased approximately four times, from 0.208 to 0.811, as heritability increased from 0.1 to 0.5 under the 5% male preselection percentage and random female preselection.

### Reliability of the GEBV in the reference population comprising both bulls and cows

The combined reliabilities in the reference population composed of 10,000 preselected bulls and 10,000 or 20,000 preselected cows are calculated by (10) and shown together with the reliabilities of reference populations composed solely of bulls or cows (Table 7). The cows in Table 7 are only those preselected on EBV from their individual records, because the combined reliability was almost equivalent whether heifers were preselected on PA or cows were preselected on the EBV from their own records. The combined reliability increased as the number of cows increased from 10,000 to 20,000, and this trend was more conspicuous for high-heritability traits (h^2^ = 0.5) than low-heritability traits (h^2^ = 0.1). As shown in Table 2 and 6, this result again confirmed cows’ favorable properties regarding the reliability of high-heritability traits. The contribution of cows in reliability of the combined population compared with that of a reference population composed of bulls only, i.e., the difference between combined reliability due to bulls and cows and the reliability due to bulls only, ranged from 0.03 to 0.22. The reliability for a reference population composed of either bulls or cows solely was computed by using (1). The number of bulls equal to a cow in terms of the reliability of the reference population created from both preselected bulls and cows computed by using (12) coincided with the number computed by using (9). That is, the number of bulls equal to a cow in regard to the reliability of GEBV in the reference population comprising both bulls and cows agreed with the number of bulls equal to a cow in the reference population created solely from bulls or cows in Table 6.

## DISCUSSION

### Benefit of cows regarding the reliability of GEBV for high-heritability traits

The superiority of the reliability of the GEBV from a bulls-only reference population over the cow population decreased as heritability increased regardless of whether animals in the reference population were preselected (Tables 1, 2). To improve GP, the same individuals should be both genotyped and phenotyped instead of genotyping parents and phenotyping their progeny [5]. In the current study, cows are both genotyped and phenotyped, whereas bulls are genotyped and their progeny are phenotyped. Because the reliability of a cow’s EBV was based on her own record, the increase in reliability concurrent with an increase in heritability was greater for cows than for bulls (Table 3). For example, the reliability of a cow’s EBV based on her own record and that of a bull’s EBV based on 50 of his daughters’ records corresponding to a heritability of 0.1 are 0.236 and 0.562; when heritability is 0.5, these are 0.604 and 0.877, respectively. Consequently, we consider that genotyping of cows with phenotypes is advantageous for high-heritability traits from the point of increasing the reliability of GEBV. The value of genotyping of cows with phenotypes was reduced by increasing the number of daughters per test bull (results not shown), because the reliability of bulls increased with increases in the number of daughters per test bull (Table 1).

### The effects of preselection on reliability and bias of GEBV

The effect of preselection on reducing the variance of G increased as heritability increased, thereby decreasing the reliability of the GEBV of preselected animals. However, the reliability of GEBV in the reference population increased as heritability increased even if preselection had been practiced (Tables 1, 2). This result indicates that the effect of the increase in heritability on the increase in the reliability of the EBV of animals by using a cow’s own record or progeny testing of bulls was greater than that on the decrease in reliability due to reduction of the variance of G from preselection.

The effect of preselection on the decreased reliability of GEBV became more deleterious for smaller reference populations (Tables 1, 2). This result is explained by (1). That is, the reliability of GEBV after preselection is written as:

$${\text{r}}_{\text{GEBV},\text{G}*}^{2}={\text{r}}_{\text{GEBV}}^{2}\frac{1-{\text{kr}}_{\text{EBV}}^{2}}{1-{\text{kr}}_{\text{EBV}}^{2}{\text{r}}_{\text{GEBV}}^{2}},$$

where r_GEBV_ is the accuracy of GEBV in the absence of preselection or under random selection. By contrast, the ratio of reliability after preselection (${\text{r}}_{\text{GEBV},\text{G}*}^{2}$) to that under random selection (${\text{r}}_{\text{GEBV}}^{2}$) is written as:

$$\frac{{\text{r}}_{\text{GEBV},\text{G}*}^{2}}{{\text{r}}_{\text{GEBV}}^{2}}=\frac{1-{\text{kr}}_{\text{EBV}}^{2}}{1-{\text{kr}}_{\text{EBV}}^{2}{\text{r}}_{\text{GEBV}}^{2}}$$

The ratio is 1.0 without loss of reliability when ${\text{r}}_{\text{GEBV}}^{2}=1.0$. The ratio becomes smaller with decreases in reliability under random selection (${\text{r}}_{\text{GEBV}}^{2}$) when the reliability of preselection (${\text{r}}_{\text{EBV}}^{2}$) and the intensity of preselection are held constant. The main factor to decrease the reliability of GEBV under random selection (${\text{r}}_{\text{GEBV}}^{2}$) is a reduction in the reference population size. Therefore, the effect of decreasing the reliability of GEBV due to preselection became much greater as the size of the reference population decreased.

Bias emerged when the cow reference population was under selection, but it decreased with increase in the size of the cow reference population (Table 5). The inclusion of cows in the reference population slightly reduced the bias in GEBV [17]. Routine genotyping of heifer calves or yearling heifers can be a cost-effective strategy for enhancing the genetic value of replacement females on commercial dairy farms [21]. That is, saying that a replacement decision was based on GEBV implies that genotyped heifer calves or yearling heifers were included in the reference population. In general, it is easier to increase reference population size by using cows than bulls. Expanding the reference population alleviated bias when heritability and intensity of preselection remained constant (Tables 4, 5). This effect occurs because the regression coefficient as a criterion of bias is defined as ($\frac{1-{\text{kr}}_{\text{EBV}}^{2}}{1-{\text{kr}}_{\text{EBV}}^{2}{\text{r}}_{\text{GEBV}}^{2}}$) as shown in (2) and because the reliability of GEBV (${\text{r}}_{\text{GEBV}}^{2}$) under random selection increases with expanding reference population size. Note that the regression coefficient as a criterion of bias is equal to the ratio of reliability after preselection (${\text{r}}_{\text{GEBV},\text{G}*}^{2}$) to that under random selection (r^2^_GEBV_), i.e., $\frac{{\text{r}}_{\text{GEBV},\text{G}*}^{2}}{{\text{r}}_{\text{GEBV}}^{2}}$ That is, both of them can be written as: $\frac{1-{\text{kr}}_{\text{EBV}}^{2}}{1-{\text{kr}}_{\text{EBV}}^{2}{\text{r}}_{\text{GEBV}}^{2}}$. Consequently, bias and reduction in reliability of GEBV due to preselection was alleviated by expanding reference population. Therefore, cows can contribute to reducing bias and increasing reliability due to their ease of use in expanding reference population size and by providing more recent animals (compared with bulls). Cows’ contribution was determined to improving GP in breeding schemes where few bulls with traditional evaluations were added annually [22,23]. Older bulls may contribute only slightly to increasing genomic reliability because of linkage decay between the validation and ancestral populations, resulting in r_g_<1.0 between bulls and cows and lowering reliability [7]. The young selection candidates are more closely related to the animals in the reference population when the reference population consists of cows or a combination of bulls and cows instead of bulls only [7]. The GP is more reliable when juvenile animals share their recent pedigree with animals in the reference population [24,25]. Cows are easier in expanding reference population size compared with bulls and alleviate bias and reduction in reliability of bulls’ GEBV due to higher preselection by expanding reference population of cows.

### The value of cows compared with that of bulls in terms of the reliability of GEBV

The overall reliability in the reference population comprising both bulls and cows was not the sum of the reliabilities from those containing bulls or cows only (Table 7); this result indicated that marker information between bulls and cows was not independent, which was in agreement with the reliability results obtained from Danish cows and US bulls [26]. That is, the off-diagonal elements in (10) derived from index selection theory for the reference populations containing both bulls and cows were not zero.

The number of bulls equal to a cow in reliability of GEBV as calculated from (12) in the reference population containing both bulls and cows agreed with the number computed from (9) in a reference population created solely from bulls or cows. This effect occurs because the increased reliability due to the addition of cows into a bulls-only population was converted to the increase per head in the bulls-only population and the numbers of bulls only and cows only to yield the increased reliability was compared. Consequently, the number of bulls equal to a cow in terms of the reliability of the combined reference population could be computed by using the simple formula of (9) applied to reference populations created solely from bulls or cows. Cows are, in general, selected randomly compared with bulls; consequently, the effect of preselection on decreased reliability and bias of GEBV would be much smaller for cows than for bulls.

### Assumption of parameters

The proportion of genetic variance explained by its markers is influenced by the effective size of the population (N_E_) and the density at which the genetic analysis covers the genome. The number of independent segments present in the genome is expected to be lower at low (compared with high) N_E_ [27]. Therefore, the accuracy of GP is expected to be higher in a population with a smaller N_E_ than in a population with a larger N_E_. An N_E_ of 750 was assumed by [8], whereas an N_E_ of 100 was assumed in the current study and by [5,20].

Simulation studies have shown that the accuracy of GEBV decreases slowly over generations when mating is random [1, 28] and more rapidly when selection is considered [29]. Given that recombination breaks up linkage disequilibrium (LD) in both situations, this finding indicates that selection is an important factor for decreasing LD between markers and qualitative trait loci. That is, accurate prediction of GEBV strongly depends on the persistence of LD between markers and qualitative trait loci across generations. However, we used a single generation in the current study to develop a simple formula for assessing the accuracy of GEBV that accounted for the effect of selection instead of accounting for persistent accuracy across generations. Advances in the use of sequence data and gene expression studies would lead to improved persistence of GP and potentially lead to greater reliabilities [12]. The development of methodology for estimating persistent accuracy of GEBV across generations is warranted.

An empirical value for the number of independent chromosome segments could be used in place of nG [6]. The accuracy of GEBV was also proposed by [27]. The current study used the original formula [6], because the number of bulls equal to a cow in terms of the reliability of GEBV must be compared under the same reliability of GEBV and is written without the term of the reliability of GEBV as shown in (9). The accuracies of the GEBV in this study are not comparable with the correlations between GEBV and degressed regression proofs determined from validation studies of field data [14,30]. The reason for this difference is that the information here is based on LD information alone, whereas the markers used for prediction of GEBV capture information on both genetic relationship and LD [24]. Both the theoretical number of bulls equal to a cow and the actual number derived from field data in terms of reliability warrants further study to validate the developed formula. However, the value of cows in terms of reliability of GEBV in the reference population under selection was simplified to the formula in (9), which likely will be a highly useful guideline for creating reference populations containing both bulls and cows.

## CONCLUSION

Bias was greater for bulls than cows, because the intensity of preselection was higher in the bull population. Bias and reduction in reliability of GEBV due to preselection was alleviated by expanding reference population and by increasing the size of the reference population of cows even if the size of the reference population of bulls was held constant. Therefore, cows can contribute to reducing bias and increasing reliability due to their ease of use in expanding reference population size and by providing more recent animals compared with bulls. The number of bulls equal to a cow in a standpoint of bringing about the same size of reliabilities of the GEBV of preselected animals in the reference population was described as a simple formula (9) composed of reliability of the EBV of the trait of interest, preselection intensity and accuracy whether a reference population is either bulls/cows only or bulls and cows both. The generalized formulas presented in this study do satisfy the property of invariance and thus, is a general guideline for creating the reference population under selection for any combination of bull and cow populations.

## Figures and Tables

**Table 1:** The reliability of GEBV of non-preselected bulls at 100% preselection and the ratio of reliability of preselected bulls at <100% preselection to that of non-preselected bulls

Heritability	No. of animals	Bulls preselected according to PA1) (progeny testing 50 daughters per test bull)			Bulls preselected according to PA2) (progeny testing 100 daughters per test bull)		

		Preselection percentage (%)					

		5	30	100	5	30	100
0.1	5,000	0.8982	0.9137	0.3189	0.8817	0.9001	0.3748
	10,000	0.9209	0.9332	0.4837	0.9111	0.9253	0.5453
	20,000	0.9452	0.9540	0.6519	0.9406	0.9503	0.7057
	40,000	0.9661	0.9716	0.7893	0.9643	0.9703	0.8275
0.3	5,000	0.8426	0.8677	0.4006	0.8346	0.8613	0.4259
	10,000	0.8823	0.9018	0.5721	0.8780	0.8986	0.5974
	20,000	0.9218	0.9352	0.7278	0.9200	0.9340	0.7479
	40,000	0.9532	0.9615	0.8425	0.9526	0.9611	0.8558
0.5	5,000	0.8039	0.8361	0.4223	0.7989	0.8322	0.4378
	10,000	0.8536	0.8789	0.5938	0.8510	0.8770	0.6090
	20,000	0.9028	0.9204	0.7452	0.9019	0.9198	0.7570
	40,000	0.9419	0.9528	0.8540	0.9417	0.9527	0.8617

GEBV, genomically enhanced breeding value; PA, parental average.

1) PA was calculated by using EBVs from sire (from 50 daughters) and dam.

2) PA was calculated by using EBVs from sire (from 100 daughters) and dam.

**Table 2:** The reliability of GEBV of cows

Heritability	No. of animals	Cows preselected according to PA1)				Cows preselected according to the EBV from their individual records			

		Preselection percentage (%)							

		70	80	90	100	70	80	90	100
0.1	5,000	0.0683	0.0699	0.0721	0.0769	0.0709	0.0720	0.0735	0.0769
	10,000	0.1279	0.1306	0.1344	0.1429	0.1325	0.1343	0.1370	0.1429
	20,000	0.2269	0.2311	0.2370	0.2500	0.2339	0.2368	0.2410	0.2500
	40,000	0.3698	0.3754	0.3832	0.4000	0.3792	0.3830	0.3883	0.4000
0.3	5,000	0.1620	0.1690	0.1789	0.2000	0.1770	0.1812	0.1871	0.2000
	10,000	0.2788	0.2891	0.3034	0.3333	0.3008	0.3068	0.3152	0.3333
	20,000	0.4361	0.4486	0.4656	0.5000	0.4625	0.4695	0.4793	0.5000
	40,000	0.6073	0.6193	0.6354	0.6667	0.6324	0.6390	0.6481	0.6667
0.5	5,000	0.2243	0.2376	0.2561	0.2941	0.2559	0.2630	0.2729	0.2941
	10,000	0.3664	0.3840	0.4077	0.4546	0.4075	0.4164	0.4288	0.4546
	20,000	0.5363	0.5549	0.5793	0.6250	0.5791	0.5880	0.6002	0.6250
	40,000	0.6981	0.7138	0.7336	0.7692	0.7334	0.7406	0.7502	0.7692

GEBV, genomically enhanced breeding valuel; PA, parental average; EBV, estimated breeding value.

1) PA was calculated by using EBVs from sire (from 50 daughters) and dam.

**Table 3:** Reliability of a bull’s progeny testing, cow’s estimated breeding value (EBV) based on her individual record and parental average (PA1)), and preselection on PA1)

Heritability	Bull’s progeny testing2)	Cow’s EBV	Preselection on PA
0.1	0.562 (1.0) 3)	0.236 (1.0)	0.165 (1.0)
0.3	0.802 (1.428)	0.447 (1.893)	0.276 (1.666)
0.5	0.877 (1.561)	0.604 (2.556)	0.344 (2.082)

1) PA was calculated by using EBVs from sire (from 50 daughters) and dam.

2) 50 daughters per test bull.

3) The figures within parentheses are the ratio of reliability to that for a heritability of 0.1.

**Table 4:** Regression coefficients of true breeding values on the GEBV of preselected bulls in the reference population1)

Heritability	No. of animals	Bulls preselected according to PA2) (progeny-testing 50 daughters per test bull)		Bulls preselected according to PA3) (progeny-testing 100 daughters per test bull)	

		Preselection percentage (%)			

		5	30	5	30
0.1	5,000	0.898	0.914	0.882	0.900
	10,000	0.921	0.933	0.911	0.925
	20,000	0.945	0.954	0.941	0.950
	40,000	0.966	0.972	0.964	0.970
0.3	5,000	0.843	0.868	0.835	0.861
	10,000	0.882	0.902	0.878	0.899
	20,000	0.922	0.935	0.920	0.934
	40,000	0.953	0.961	0.953	0.961
0.5	5,000	0.804	0.836	0.799	0.832
	10,000	0.854	0.879	0.851	0.877
	20,000	0.903	0.920	0.902	0.920
	40,000	0.942	0.953	0.942	0.953

GEBV, genomically enhanced breeding valuel; PA, parental average; EBV, estimated breeding value.

1) In all cases when the preselection percentage was 100%, the regression coefficient was 1.0.

2) PA was calculated by using EBVs from sire (from 50 daughters) and dam.

3) PA was calculated by using EBVs from sire (from 100 daughters) and dam.

**Table 5:** Regression coefficients of true breeding values on the GEBV of preselected cows in the reference population1)

Heritability	No. of animals	Cows preselected according to the EBV of their individual records		Cows preselected according to PA2)	

		Preselection percentage (%)			

		70	90	70	90
0.1	5,000	0.888	0.937	0.922	0.956
	10,000	0.896	0.941	0.927	0.959
	20,000	0.907	0.948	0.936	0.964
	40,000	0.925	0.958	0.948	0.971
0.3	5,000	0.810	0.894	0.885	0.935
	10,000	0.837	0.910	0.902	0.946
	20,000	0.872	0.931	0.925	0.959
	40,000	0.911	0.953	0.949	0.972
0.5	5,000	0.762	0.871	0.870	0.928
	10,000	0.806	0.897	0.897	0.943
	20,000	0.858	0.927	0.927	0.960
	40,000	0.908	0.954	0.953	0.975

GEBV, genomically enhanced breeding valuel; EBV, estimated breeding value; PA, parental average.

1) In all cases when the preselection percentage was 100%, the regression coefficient was 1.0.

2) PA was calculated by using EBVs from sire (from 50 daughters) and dam.

**Table 6:** The number of bulls equal to a cow in regard to bringing about the same size of reliability of GEBV of preselected animals in the reference population

Heritability	Preselection (%) of bulls according to PA1)	Preselection (%) of cows according to the EBV of their individual records		Preselection (%) of cows according to PA1)	

		Preselection percentage of cows (%)			

		70	100	70	100
0.1	5	0.183	0.208	0.190	0.208
0.1	30	0.178	0.203	0.186	0.203
0.3	5	0.379	0.490	0.422	0.490
0.3	30	0.363	0.469	0.403	0.469
0.5	5	0.562	0.811	0.669	0.811
0.5	30	0.530	0.763	0.630	0.763

GEBV, genomically enhanced breeding valuel; PA, parental average; EBV, estimated breeding value.

1) PA was calculated by using EBVs from sire (from 50 daughters) and dam.

**Table 7:** Reliability of GEBV of the mixed reference population comprising preselected bulls and cows

Heritability	No. of cows	Preselection percentage (%) bulls - cows	Reliability of bulls only (progeny-testing 100 daughters per test bull)	Reliability of cows only	Reliability
0.1	10,000	5 – 70	0.497	0.126	0.531
	10,000	5 – 100	0.497	0.143	0.536
	10,000	30 – 70	0.505	0.126	0.538
	10,000	30 – 100	0.505	0.143	0.542
	20,000	5 – 70	0.497	0.223	0.560
	20,000	5 – 100	0.497	0.250	0.569
	20,000	30 – 70	0.505	0.223	0.566
	20,000	30 – 100	0.505	0.250	0.575
0.3	10,000	5 – 70	0.524	0.277	0.598
	10,000	5 – 100	0.524	0.333	0.616
	10,000	30 – 70	0.537	0.277	0.607
	10,000	30 – 100	0.537	0.333	0.624
	20,000	5 – 70	0.524	0.434	0.652
	20,000	5 – 100	0.524	0.500	0.670
	20,000	30 – 70	0.537	0.434	0.658
	20,000	30 – 100	0.537	0.500	0.683
0.5	10,000	5 – 70	0.518	0.365	0.623
	10,000	5 – 100	0.518	0.455	0.656
	10,000	30 – 70	0.534	0.365	0.633
	10,000	30 – 100	0.534	0.455	0.664
	20,000	5 – 70	0.518	0.535	0.690
	20,000	5 – 100	0.518	0.625	0.733
	20,000	30 – 70	0.534	0.535	0.697
	20,000	30 – 100	0.534	0.625	0.738

GEBV, genomically enhanced breeding valuel; EBV, estimated breeding value.

Cows were preselected according to the EBV of their individual records.

Bulls were preselected on PA calculated by using EBVs from sire (from 100 daughters) and dam.

No. of bulls in the mixed reference population was 10,000.
